# Validation and comparison of non-invasive prediction models based on liver stiffness measurement to identify patients who could avoid gastroscopy

**DOI:** 10.1038/s41598-020-80136-0

**Published:** 2021-01-08

**Authors:** Youwen Hu, Zhili Wen

**Affiliations:** Department of Gastroenterology, The Second Affiliated Hospital of Nanchang University, No.1 Minde Rd, Nanchang, 330006 China

**Keywords:** Liver diseases, Liver cirrhosis, Portal hypertension

## Abstract

Several non‐invasive tests (NITs) based on liver stiffness measurement (LSM) have been developed to rule out varices needing treatment (VNT), including the Baveno VI criteria (B6C), the expanded Baveno VI criteria (EB6C), the LSM-spleen diameter to platelet ratio score (LSPS), and the VariScreen algorithm. We aimed to validate and compare those NITs in patients with compensated advanced chronic liver disease (cACLD). This retrospective study enrolled 354 patients with cACLD; LSM, platelet count (PLT), international normalized ratio (INR), gastroscopy and spleen diameter (SD) were collected. VNT prevalence was 28.5%. In comparison, patients with VNT included higher LSM, INR, and SD and lower PLT. Gastroscopies were spared for 27.7% of patients using the B6C with 1.0% VNT missed rate, 47.2% of patients using the EB6C with 5.9% VNT missed rate, 57.6% of patients using the LSPS with 9.9% VNT missed rate, and 45.5% of patients using the VariScreen algorithm with 3.0% VNT missed rate. Only the B6C and the VariScreen algorithm could safely avoid gastroscopies, and the VariScreen algorithm spared more gastroscopies than the B6C. The results were consistent with the previous when performed subgroup analysis. In conclusion, the VariScreen algorithm performed the best and can be used in clinical.

## Introduction

Portal hypertension (PH) is defined as abnormally increased pressure in the portal venous system, and includes prehepatic, intrahepatic, and posthepatic portal hypertension. It is a common complication of chronic liver disease (CLD), especially cirrhosis^1^. However, direct portal pressure measurement requires cannulation of the portal or umbilical veins, which is invasive and difficult to conduct. Alternatively, portal hypertension can be evaluated by the hepatic venous pressure gradient (HVPG)^2^. The HVPG is calculated as the wedged hepatic vein pressure minus the free hepatic vein pressure, and typically ranges from 3 to 5 mmHg. Portal hypertension is indicated when the HVPG > 5 mmHg, while a HVPG ranging from 5 to 9 mmHg indicates subclinical portal hypertension. Collateral circulation occurs when the HVPG ≥ 10 mmHg, termed clinically significant portal hypertension (CSPH), and the most common consequence of collateral circulation is esophageal varices (EV). Variceal bleeding can occur when the HVPG ≥ 12mmHg^3,4^, which is potentially life-threatening, and the 6-week mortality is as high as nearly 18%^5^. Therefore, the early diagnosis of varices needing treatment (VNT) is crucial.

Liver cirrhosis is a pathological diagnosis that includes compensated and decompensated stages. However, compensated cirrhosis, especially stage Ia, is difficult to diagnose with only clinical and laboratory examinations, it typically requires liver biopsy^6^. Liver biopsy is an invasive procedure that can cause rare but potentially life-threatening complications, and improper sampling can lead to a false-negative result. Additionally, there may be PHT during liver fibrosis. Therefore, it is necessary to screen for EV in patients with compensated advanced chronic liver disease (cACLD), not just liver cirrhosis. Gastroscopy is the gold standard for the diagnosis of EV. While, due to the invasiveness, discomfort and exorbitant cost, many patients with cACLD refuse to undergo gastroscopy. Therefore, NITs for safely excluding VNT is of imperative necessity.

Several NITs have been developed to rule out VNT, including platelet/MELD strategy^7^, the platelet-albumin criteria^8^, the LGV diameter-SS to PLT ratio index (LSPI)^9^, Platelet count/spleen diameter ratio (PSR)^10^, liver stiffness measurement (LSM)^11,12^, spleen stiffness measurement (SSM)^13,14^, the Baveno VI criteria (B6C)^15^, the expanded Baveno VI criteria (EB6C)^16^, LSM-spleen diameter (SD) to platelet ratio score (LSPS)^17^, and the VariScreen algorithm^18^, etc. Transient elastography (TE) has been proven to be an accurate and noninvasive technique for assessing hepatic fibrosis and liver cirrhosis^19^. The B6C was proposed in the 2015 Baveno VI consensus^20^. It recommends that when LSM < 20 kPa and platelet count (PLT) > 150,000/L, the possibility of VNT is very low, and gastroscopies can be safely avoided, and the EB6C expands the LSM threshold to 25 kPa and the PLT threshold to 110,000/L. The LSPS was proposed by Kim et al.^17^, and the formula was LSM*SD /PLT. They suggested that gastroscopy could be safely avoided when the score was less than 3.5. However, their study population only comprised patients with hepatitis B cirrhosis. Subsequently, Lee et al.^21^ expanded the study population to patients with cACLD, and identified that the optimal cutoff value for ruling out VNT was 1.47. The VariScreen algorithm was a sequential algorithm which incorporated data on PLT or LSM, adjusted for etiology, gender, and international normalized ratio (INR). It is available as a free calculator (http://forge.info.univ-angers.fr/~gh/wstat/pler-please-variscreen.php). Currently, LSM has been widely used in patients with CLD in clinical practice, and several NITs based on LSM has been developed to rule out VNT. However, some NITs still need to further validate the effectiveness and safety. Moreover, external validation is required for comparison between these NITs. The B6C, EB6C, LSPS, and VariScreen algorithm are all NITs based on LSM, and our purpose of the retrospective study is to validate and compare those four NITs in patients with cACLD of different etiologies.

## Results

### Baseline characteristics of study populations

A total of 366 patients with cACLD were enrolled in our study. The median age was 48 years, and about 70% were men, nearly four-fifth were cirrhosis. HBV related cACLD was the most frequent etiology, accounting for 80.1%, followed by alcoholic cACLD and non-alcoholic fatty liver disease (NAFLD). A majority of patients with HBV related cACLD received oral antiviral treatment. The median LSM, PLT, SD and INR were 15.5 kPa, 114,500/ul, 13.0 cm, and 1.09, respectively. VNT prevalence was 28.5%. Patients with VNT showed higher LSM, longer SD, worse INR, and lower PLT. There was no difference in age, gender, etiology, and examination time interval between patients with and without VNT. The detailed demographic and clinical characteristics of the patients were summarized in Table 1.

**Table 1:** Baseline characteristics of the study populations.

	Total (n = 354)	Non-VNT (n = 253)	VNT (n = 101)	*P*
Age (years)	48 (47, 55)	48 (46, 57)	48 (47, 53)	0.58
Male, n (%)	249 (70.3)	177 (70.0)	72(69.3)	0.81
**Etiology**				0.03
Virus	293	218	75	
Alcohol	31	17	14	
NAFLD	30	18	12	
Cirrhosis, n (%)	271 (76.6)	175 (69.2)	96 (95.0)	< 0.001
**Receiving antiviral treatment**				
Yes	220	146	74	< 0.001
No	73	72	1	
Time between gastroscopy and TE (day)	4 (2, 40)	4 (2, 43.5)	5 (2, 13)	0.64
Time between ultrasonography and TE (day)	5 (1, 88)	4 (1, 99)	36 (4, 61)	0.07
Time between PLT and TE (day)	3 (2, 4)	3 (2, 4)	3 (2, 4)	0.12
Time between INR and TE (day)	3 (2, 4)	3 (2, 4)	3 (2, 4)	0.12
LSM (Kpa)	15.5(12.9, 25.8)	14.1 (12.4, 20.8)	25.7 (17.4, 41.3)	< 0.001
PLT (10^3/ul)	114.5 (73, 173.5)	142.0 (104.5, 187.0)	62.0 (48.5, 87.5)	< 0.001
SD (cm)	13.0 (12.2, 14)	12.9 (12, 13.6)	13.3 (12.6, 14.2)	< 0.001
INR	1.09 (1.01, 1.19)	1.05 (0.98, 1.14)	1.18 (1.12, 1.26)	< 0.001

VNT, varices needing treatment; TE, transient elastography; PLT, platelet count; LSM, liver stiffness measurement; SD, spleen diameter; INR, international normalized ratio.

### Performance and safety of the NITs in patients with cACLD

**Figure 1:**
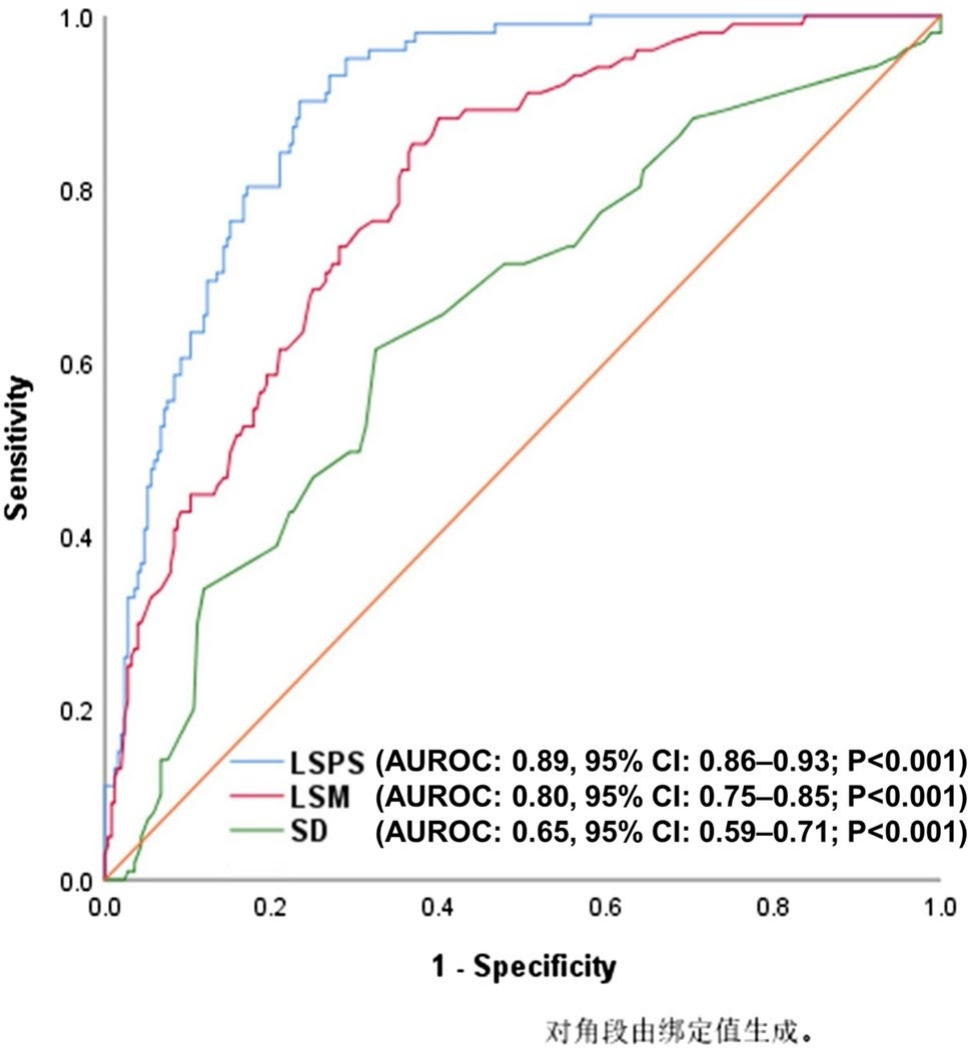
ROC curve of LSPS, LSM and SD; LSPS, liver stiffness-spleen size-to-platelet ratio risk score; LSM, liver stiffness measurement; SD, spleen diameter; AUROC, area under the receiver-operating characteristic curve.

The optimal cut-off value was 2.57 for LSPS to rule out VNT. The area under the receiver-operating characteristic curve (AUROC) of the LSPS was 0.89 [95% confidence interval (CI) 0.86–0.93; *P* < 0.001], which was better than LSM (AUROC, 0.80, 95% CI 0.75–0.85; *P* < 0.001) and SD (AUROC, 0.65, 95% CI 0.59–0.71; *P* < 0.001) (Fig. 1). The number of patients with fulfilled and unfulfilled of the four NITs was displayed in Table 2. Compared with patients who did not fulfill the NITs, those who fulfilled the NITs had a lower risk of developing VNT (Table 2).

**Table 2:** Number of non-VNT and VNT generated by the NITs in patients with cACLD.

Variables	Total (n = 354)	Non-VNT (n = 253)		VNT (n = 101)
		No varix (n = 215)	Small varices (n = 38)	
**B6C**				
Within	98	94	3	1
Outside	256	121	35	100
**EB6C**				
Within	167	149	12	6
Outside	187	66	26	95
**LSPS**				
Within	204	179	15	10
Outside	150	36	23	91
**VariScreen**				
Within	161	145	13	3
Outside	193	70	25	98

NITs, non-invasive tests; VNT, varices needing treatment; cACLD, compensated advanced chronic liver disease; B6C, Baveno VI criteria; EB6C, Expanded Baveno VI criteria; LSPS, liver stiffness-spleen diameter to platelet ratio risk score.

The sensitivity, specificity, positive predictive value (PPV), negative predictive value (NPV), positive likelihood ratio (PLR), negative likelihood ratio (NLR), and diagnostic accuracy of the four NITs were shown in Table 3. The B6C had highest sensitivity and NPV, and the LSPS had the highest specificity, PPV, PLR and diagnostic accuracy. Gastroscopies were spared for 27.7% (98/354) of patients using the B6C [VNT missed in 1.0% (1/101)], 47.2% (167/354) of patients using the EB6C [VNT missed in 5.9% (6/101)], 57.6% (204/354) of patients using the LSPS [VNT missed in 9.9% (10/101)], and 45.5% (161/354) of patients using the VariScreen algorithm [VNT missed in 3.0% (3/101) ] (Table 3). The EB6C and the LSPS could spare more gastroscopies than the B6C and The VariScreen algorithm, while, based on the VNT missed rate ≤ 5%, only the B6C and the VariScreen algorithm could safely avoid unnecessary gastroscopies. In particular, the VNT missed rate of the LSPS significantly higher than the rates of the other three NITs (Table 3).

**Table 3:** Performance and safety of the NITs in patients with cACLD.

	B6C	EB6C	LSPS	VariScreen
Sensitivity (%)	99.0	94.1	90.1	97.0
Specificity (%)	38.3	63.6	76.7	62.5
PPV (%)	39.1	50.8	60.7	50.8
NPV (%)	98.9	96.4	95.1	98.1
PLR	1.61	2.59	3.86	2.58
NLR	0.03	0.09	0.13	0.05
Diagnostic accuracy (%)	55.6 (197/354)	72.3 (256/354)	80.5 (285/354)	72.3 (256/354)
Spared gastroscopy (%)	27.7 (98/354)	47.2 (167/354)	57.6 (204/354)	45.5 (161/354)
Missed VNT (%)	1.0 (1/101)	5.9 (6/101)	9.9 (10/101)	3.0 (3/101)

NITs, non-invasive tests; cACLD, compensated advanced chronic liver disease; B6C, Baveno VI criteria; EB6C, Expanded Baveno VI criteria; LSPS, liver stiffness-spleen diameter to platelet ratio risk score; PPV, Positive Predictive Value; NPV, Negative Predictive Value; PLR, Positive Likelihood Ratio; NLR, Negative Likelihood Ratio; VNT, varices needing treatment.

### Performance and safety of the NITs in subgroups

**Figure 2:**
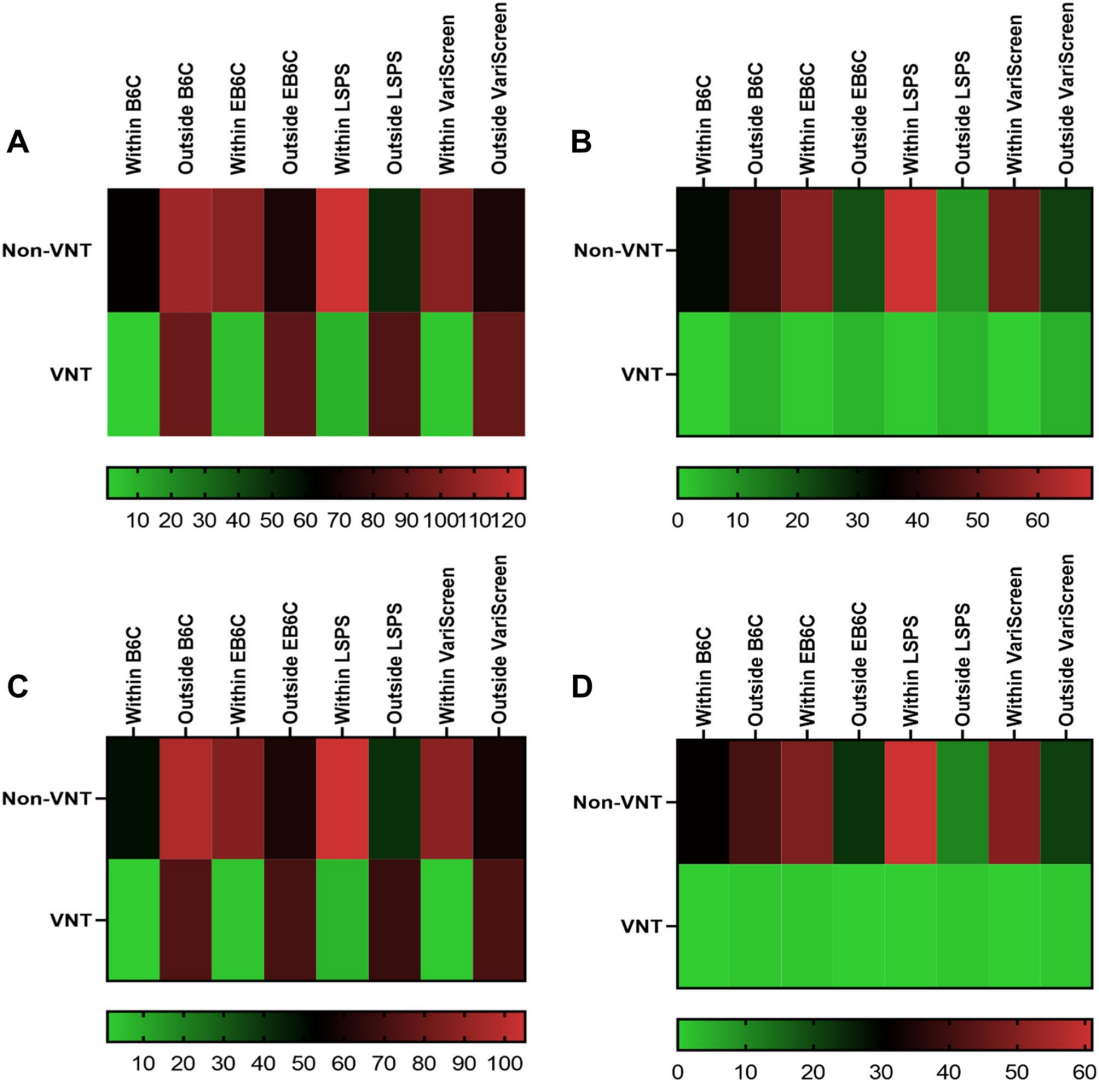
Heat map of subgroups. (**A**) cirrhotic patients; (**B**) Non-cirrhotic patients; (**C**) patients receiving antiviral treatment; (**D**) patients non-receiving antiviral treatment; VNT, varices needing treatment Imaging software: GraphPad Prism (version 7; GraphPad Software Inc., San Diego, CA): https://www.graphpad.com/scientific-software/prism/.

Considering the complexity of the patients included in the study, we conducted subgroup analysis to avoid the interference of confounding factors. There were 271 cirrhotic patients and 83 non-cirrhotic patients. In the cirrhosis group, gastroscopies were spared for 24.0% of patients (VNT missed in 1.0%), 40.2% of patients (VNT missed in 5.2%), 49.4% of patients (VNT missed in 9.4%), and 39.5% of patients (VNT missed in 3.1%) when applied the B6C, the EB6C, the LSPS, and the VariScreen algorithm, respectively. In the non-cirrhosis group, gastroscopies were spared for 39.8% of patients (VNT missed in 0%), 69.9% of patients (VNT missed in 20%), 84.3% of patients (VNT missed in 20%), and 65.1% of patients (VNT missed in 0%) when applied the B6C, the EB6C, the LSPS, and the VariScreen algorithm, respectively. There were 293 patients with HBV related cACLD in our study, and 220 patients had received oral antiviral treatment. In the receiving oral antiviral treatment group, gastroscopies were spared for 22.7% of patients (VNT missed in 1.4%), 40.5% of patients (VNT missed in 4.1%), 50.9% of patients (VNT missed in 9.5%), and 40.9% of patients (VNT missed in 2.7%) when applied the B6C, the EB6C, the LSPS, and the VariScreen algorithm, respectively. In the non-receiving oral antiviral treatment group, gastroscopies were spared for 42.5% of patients (VNT missed in 0%), 68.5% of patients (VNT missed in 100%), 83.6% of patients (VNT missed in 0%), and 69.9% of patients (VNT missed in 0%) when applied the B6C, the EB6C, the LSPS, and the VariScreen algorithm, respectively (Table 4). The numbers of fulfilled and unfulfilled of those NITs in subgroups were represented by heat map (Fig. 2), and the detailed data were shown in the supplementary materials (Table S1–S4).

**Table 4:** Performance and safety of the NITs in subgroups.

	Spared gastroscopy (%)				Missed VNT (%)			
	B6C	EB6C	LSPS	VariScreen	B6C	EB6C	LSPS	VariScreen
Cirrhosis	24.0 (65/271)	40.2 (109/271)	49.4 (134/271)	39.5 (107/271)	1.0 (1/96)	5.2 (5/96)	9.4 (9/96)	3.1 (3/96)
Non-cirrhosis	39.8 (33/83)	69.9 (58/83)	84.3 (70/83)	65.1 (54/83)	0 (0/5)	20 (1/5)	20 (1/5)	0 (0/5)
Receiving antiviral treatment	22.7 (50/220)	40.5 (89/220)	50.9 (112/220)	40.9 (90/220)	1.4 (1/74)	4.1 (3/74)	9.5 (7/74)	2.7 (2/74)
Non-receiving antiviral treatment	42.5 (31/73)	68.5 (50/73)	83.6 (61/73)	69.9 (51/73)	0 (0/1)	100 (1/1)	0 (0/1)	0 (1/1)

NITs, non-invasive tests; VNT, varices needing treatment; B6C, Baveno VI criteria; EB6C, Expanded Baveno VI criteria LSPS, liver stiffness-spleen diameter to platelet ratio risk score.

## Discussion

CLD is a common and frequently occurring disease. Once it develops into the decompensated stage, the mortality increases significantly. Among them, PH and gastroesophageal varices are common complications of CLD. In our study, VNT prevalence was 28.5% in patients with cACLD, which is higher than those reported in literature (13.0%-20.9%)^13,18,21–23^. We think it may be related to the sample size. Considering the prevalence and risk of VNT, early diagnosis is necessary. Due to the invasiveness, discomfort and exorbitant cost of gastroscopy, NITs for ruing out VNT safely and accurately is therefore urgently needed in clinics. TE is a non-invasive technology for assessing the presence of liver fibrosis or cirrhosis in patients with CLD by measuring liver stiffness, which has been widely used in clinical diagnosis and treatment^24–28^. Our study showed that LSM in patients with VNT was higher than patients without VNT, which was consistent with those reported in literature. The AUROC of LSM for ruling out VNT was 0.80. Many studies have shown that LSM is effective at predicting CSPH and gastroesophageal varices^29–32^. The Baveno VI consensus recommended avoiding gastroscopy when LSM < 20 kPa and PLT > 150,000/L. Our study also validated the effectiveness and safety of the B6C, which was consistent with other published literatures^23,33–35^. However, the number of patients who can benefit from the B6C was still small, and our results shown that it was only 27.7%. Using the EB6C for ruling out VNT could enable more patients to avoid unnecessary gastroscopy. Our study showed that it could spare 19.5% more gastroscopies than using the B6C. While, the VNT missed rate was 5.9%, which meant it was not safe enough, which reflecting those previous studies^18,36^. The AUROC of the LSPS was 0.89 in our study, and could significantly spare more gastroscopies than the other three NITs. But similar to the EB6C, the LSPS was not safe either due to a high VNT missed rate (9.9%)^37^. Gastroscopies were spared for 45.5% of patients when using the VariScreen algorithm for ruling out VNT in patients with cACLD, and the VNT missed rate was 3.0%, which meant it was safe to be applied^18^.

Considering that the population enrolled in our study was cACLD, and patients with non-cirrhosis accounted for 23.4%, so we divided the population into cirrhosis and non-cirrhosis groups for a subgroup analysis. In non-cirrhotic patients, the results were consistent with those in patients with cACLD, and the VNT missed rate of the EB6C and the LSPS were up to 20%, we think it may due to only five patients with VNT in the group. Likewise, in cirrhotic patients, only the B6C and the VariScreen algorithm was safe, and the results were consistent with the previous. In our study, HBV cACLD accounted for 82.8%, and 75.1% of patients received antiviral treatment. Previous antiviral treatment is a putative confusing factor for elastography measurements. To eliminate the potential impact, we divided the patients into groups of those receiving antiviral treatment and those not receiving antiviral treatment, and we observed the similar results.

Our study has some limitations. First, it was a single-center retrospective study and, as such, a selection bias may affect the results of our research. And, the VNT's prevalence of our study was indeed higher than those reported in literature for patients with cACLD. We thought it may be due to the small sample size. However, we believed that this will not affect the validation and comparison between those models. Second, TE, gastroscopy, abdominal ultrasound, and routine blood tests were not performed on the same day. But there was no statistical difference in examination time interval between patients with and without VNT. Therefore, we did not think it could affected the results. Third, liver cirrhosis was diagnosed using laboratory, radiological, and physical examination only. Since biopsy was not performed, there may have been a misclassification of early cirrhosis as non-cirrhosis. However, our population included patients with cACLD, which would not affect the final results. Fourth, as long as oral antiviral drugs were recorded in the electronic medical record system, it was considered that patients received antiviral treatment, regardless of the duration of treatment or whether the medication was interrupted or not. Furthermore, we did not include a HBV-DNA test result, which may have affected the results. Further prospective studies are therefore needed to confirm our findings. Fifth, it was difficult to accurately assess the use of non-selective beta blockers (NSBB) for patients, moreover, the use of NSBB do not prevent the progression of EV. Therefore, we did not include the treatment of NSBB as a parameter into our study.

In conclusion, only the B6C and the VariScreen algorithm can safely to rule out VNT (VNT missed rate < 5%) in patients with cACLD, but the EB6C and the LSPS was unsafe due to the high VNT missed rate, and the VariScreen algorithm performed better than the B6C in term of the spared gastroscopy rate. therefore, we considered the VariScreen algorithm was the best in the four NITs and could be used in clinical practice.

## Methods

### Patients

This was a single-center, retrospective, cross-sectional study. The study complied the Declaration of Helsinki. All procedures were performed in accordance with relevant guidelines. This study was approved by the Ethics Committee of the Second Affiliated Hospital of Nanchang University and the written informed consent was waived due to its retrospective nature. Between June 2018 and June 2019, we retrospectively enrolled 8,683 patients who had undergone 10,882 TE exams at the Second Affiliated Hospital of Nanchang University, China. Liver cirrhosis was diagnosed using laboratory, radiological, and physical examination, and cACLD was defined as CLD with LSM ≥ 10 kPa and Child–Pugh class A^20^.

**Figure 3:**
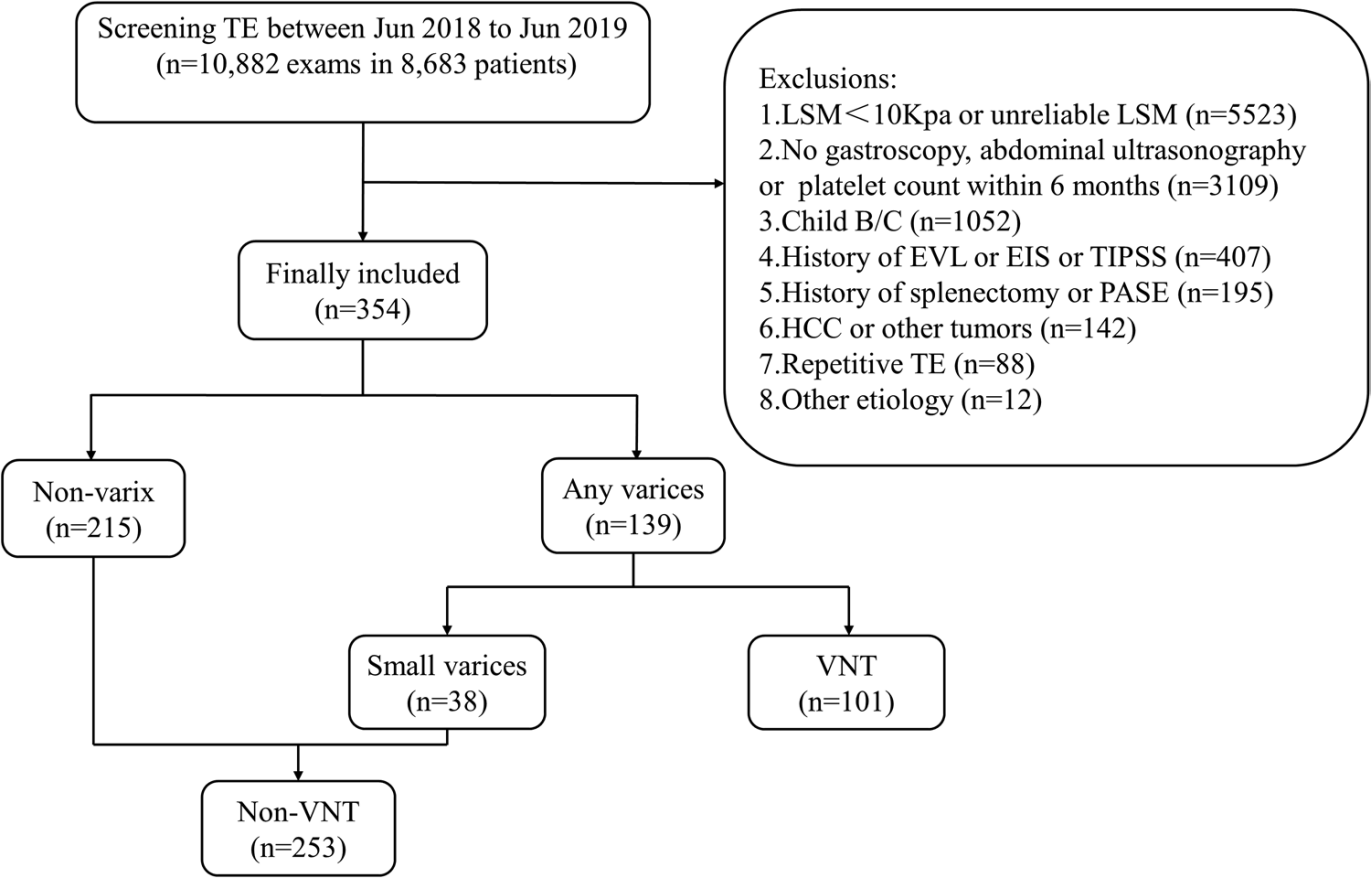
Flow chart of the study populations. TE, transient elastography; LSM, liver stiffness measurement; PLT, platelet count; INR, international normalized ratio; SD, spleen diameter; EVL, endoscopic variceal ligation; EIS, endoscopic injection sclerotherapy; TIPSS, transjugular intrahepatic portosystemic shunt surgery; PSE, partial splenic embolization; HCC, hepatocellular carcinoma; VNT, varices needing treatment.

The inclusion criterion was CLD with performance of a TE exam. Exclusion criteria were as follows: (1) LSM < 10 kPa or unreliable LSM; (2) no gastroscopy, PLT, INR and SD within 6 months; (3) Child–Pugh class B or C; (4) history of endoscopic variceal ligation, endoscopic injection sclerotherapy, or transjugular intrahepatic portosystemic shunt surgery; (5) history of splenectomy or partial splenic embolization; (6) hepatocellular carcinoma or other tumors; (7) repetitive TE; and (8) Other etiologies than virus, alcohol and NAFLD. Finally, 354 patients were enrolled retrospectively in our study (Fig. 3).

### Clinical and laboratory parameters

LSM was evaluated using TE by Fibroscan (Echosens, Paris, France). All examinations were performed by one ultrasound physician with experience of over 500 examinations. All measurements were taken with the M probe. The examination procedure was as follows: (1) patients were placed in the supine position with maximal abduction of the right arm; (2) the probe was placed level with the right lobe of the liver through an intercostal space; (3) with the assistance of ultrasound time-motion images, the operator located a portion of the liver that was at least 6 cm thick and without an extensive vascular network; (4) a reliable LSM was taken, meeting the following conditions: at least 10 valid measurements taken with a success rate ≥ 60% and an interquartile range to median ratio of < 0.3^38^.

All patients underwent gastroscopy to determine the presence and degree of EV. Gastroscopy screening was performed by experienced endoscopic physicians. The classification of EV was as follows: F1 (small varices), straight and small-caliber varices covering less than one-third of the lumen; F2 (medium varices), moderately enlarged and beaded varices; and F3 (large varices), markedly enlarged, nodular, or tumor-shaped varices occupying more than one-third of the lumen^39,40^. F2, F3, or any EV with red color signs were defined as VNT, which significantly increased the risk of hemorrhage and required treatment.

Similarly, abdominal ultrasound examination was performed by experienced operators. SD was assessed as spleen bipolar diameter. All data, including PLT, INR, gender, age, etiology, and past medical history were obtained from the electronic medical record system.

### Statistical analysis

Continuous variables with a normal distribution were expressed as the mean ± standard deviation and compared using Student’s t-test. Continuous variables with a skewed distribution were expressed as the median and interquartile range and compared using the Mann–Whitney U test. Normality test (Kolmogorow–Smironov, KS) was used to verify whether continuous variables conform to normal distribution. Categorical variables were expressed as a frequency and percentage and compared using χ^2^ or Fisher’s exact tests. The sensitivity, specificity, PPV, NPV, PLR, NLR, diagnostic accuracy and spared gastroscopy rate were calculated to assess the effectiveness of the NITs, and we chose the total number of VNT as a denominator to calculate the VNT missed rate according to the VNT-based definition^41^. ROC curves were constructed using the software MedCalc (MedCalc Software, Belgium); Heat map was drawn by GraphPad Prism (version 7; GraphPad Software Inc., San Diego, CA). Statistical analyses were performed using the software Stata V.14.0 (STATA Corp., College Station, TX, USA), and a two-tailed *P* value < 0.05 was defined as significant.

## Supplementary Information

Supplementary Information.

## Data Availability

The datasets generated during and/or analysed during the current study are available from the corresponding author on reasonable request.
