# Supplementary material for: Validation and comparison of non-invasive prediction models based on liver stiffness measurement to identify patients who could avoid gastroscopy
Source: Sci Rep. 2021 Jan 8;11:150. doi: 10.1038/s41598-020-80136-0 (PMC7794348; doi:10.1038/s41598-020-80136-0)
Supplement: Supplementary file 1 — Supplementary Information. [file 41598_2020_80136_MOESM1_ESM.docx]

**Validation and comparison of non-invasive tests based on liver stiffness measurement to identify patients who could avoid gastroscopy**

**Authors:** Youwen Hu^1^, Zhili Wen^1, *^

| Table S1: Number of non-VNT and VNT generated by the NITs in cirrhotic patients | | | |
| --- | --- | --- | --- |
| Variables | Total  (n=271) | Non-VNT  (n=175) | VNT  (n=96) |
| B6C  Within  Outside | 65  206 | 64  111 | 1  95 |
| EB6C  Within  Outside | 109  162 | 104  71 | 5  91 |
| LSPS  Within  Outside | 134  137 | 125  50 | 9  87 |
| VariScreen  Within  Outside | 107  162 | 104  71 | 3  93 |
| VNT, varices needing treatment; B6C, Baveno VI criteria; EB6C, Expanded Baveno VI criteria; LSPS, liver stiffness-spleen diameter to platelet ratio risk score | | | |

| Table S2: Number of non-VNT and VNT generated by the NITs in non-cirrhotic patients | | | |
| --- | --- | --- | --- |
| Variables | Total  (n=83) | Non-VNT  (n=78) | VNT  (n=5) |
| B6C  Within  Outside | 33  50 | 33  45 | 0  5 |
| EB6C  Within  Outside | 58  25 | 57  21 | 1  4 |
| LSPS  Within  Outside | 70  13 | 69  9 | 1  4 |
| VariScreen  Within  Outside | 54  29 | 54  24 | 0  5 |
| VNT, varices needing treatment; NITs, non-invasive tests; B6C, Baveno VI criteria; EB6C, Expanded Baveno VI criteria; LSPS, liver stiffness-spleen diameter to platelet ratio risk score | | | |

| Table S3: Number of non-VNT and VNT generated by the NITs in patients receiving antiviral treatment | | | |
| --- | --- | --- | --- |
| Variables | Total  (n=220) | Non-VNT  (n=146) | VNT  (n=74) |
| B6C  Within  Outside | 50  170 | 49  97 | 1  73 |
| EB6C  Within  Outside | 89  131 | 86  60 | 3  71 |
| LSPS  Within  Outside | 112  108 | 105  41 | 7  67 |
| VariScreen  Within  Outside | 90  130 | 88  58 | 2  72 |
| VNT, varices needing treatment; NITs, non-invasive tests; B6C, Baveno VI criteria; EB6C, Expanded Baveno VI criteria; LSPS, liver stiffness-spleen diameter to platelet ratio risk score | | | |

| Table S4: Number of non-VNT and VNT generated by the NITs in patients non-receiving antiviral treatment | | | |
| --- | --- | --- | --- |
| Variables | Total  (n=73) | Non-VNT  (n=72) | VNT  (n=1) |
| B6C  Within  Outside | 31  42 | 31  41 | 0  1 |
| EB6C  Within  Outside | 50  23 | 49  23 | 1  0 |
| LSPS  Within  Outside | 61  12 | 61  11 | 0  1 |
| VariScreen  Within  Outside | 51  22 | 51  21 | 0  1 |
| VNT, varices needing treatment; NITs, non-invasive tests; B6C, Baveno VI criteria; EB6C, Expanded Baveno VI criteria; LSPS, liver stiffness-spleen diameter to platelet ratio risk score | | | |
